# Special Issue “Synthesis of TiO_2_ Nanoparticles and Their Catalytic Activity”

**DOI:** 10.3390/nano13182544

**Published:** 2023-09-12

**Authors:** Wei Zhou

**Affiliations:** Shandong Provincial Key Laboratory of Molecular Engineering, School of Chemistry and Chemical Engineering, Qilu University of Technology (Shandong Academy of Sciences), Jinan 250353, China; wzhou@qlu.edu.cn

The current advances in the development of technologies for solar light utilization are largely due to the environmental and energy crisis caused by the rapid consumption of fossil fuels, and consequently, various applications have been implemented in domestic heating devices, the field of spaceflight, vehicles with clean energy, self-cleaning devices, the bio-pharmaceutical field, etc. To efficiently utilize abundant solar energy, different light harvesting technologies such as solar–thermal conversion, solar cells, solar focusing light, photocatalytic water splitting, CO_2_ conversion, organic synthesis, etc., have been developed as promising alternatives. In recent decades, research on solar energy conversion has developed rapidly, especially on the use of titanium dioxide (TiO_2_) as photocatalysts, resulting in a remarkable increase in energy conversion efficacy and photocatalytic performance. Besides photocatalytic energy conversion, TiO_2_ can also be used in bioactive coatings for bioimplant applications to effectively promote the formation of reactive oxygen species for the enhancement of antibacterial activity. Thus, the advances in catalytic applications using TiO_2_ nanomaterials could facilitate the utilization of photocatalysts in real conditions.

This Special Issue contains 10 research and review articles showcasing the latest developments in the synthesis of black TiO_2_, defective TiO_2_, rutile TiO_2_, and TiO_2_-based heterojunctions for the applications of photocatalytic water splitting, environmental remediation, antibacterial applications, and bioimplants, which could shed light on the challenges, opportunities, and research trends of TiO_2_-based nanoparticles and their catalytic activity.

Eight research publications amassed in this issue showcase the work of researchers from around the globe seeking to provide fundamental insights into the synthesis and photocatalytic applications of TiO_2_-based materials. To boost the photocatalytic tetracycline degradation efficiency, Hu et al. prepared Ti_2_O_3_@TiO_2_ core–shell heterojunctions via a facile heat treatment process that led to a 2.5-fold improvement compared with the pristine Ti_2_O_3_ [1]. The enhanced photocatalytic activity was ascribed to the formation of the built-in electric field in the heterojunction interface, which can be regulated by altering the ratio of Ti_2_O_3_/TiO_2_ [1]. To effectively increase the photogenerated charge carrier separation efficiency, Wang et al. presented a simple hydrothermal treatment to construct TiN@anatase-TiO_2_/rutile-TiO_2_ Z-type composites with boosted plasmonic effect [2]. Under visible light irradiation, the as-synthesized heterojunctions significantly enhanced the photodegradation activity of rhodamine B, thereby providing the possibility of the removal of photocatalytic pollutants using solar light [2]. In another study, Alyami reported an ultra-violet-assisted scalable method to construct TiO_2_ photocatalysts with abundant oxygen vacancies for photocatalytic dye removal [3]. The defective TiO_2_ with Ti^3+^ exhibited stronger visible light absorption and much higher removal efficiency of methylene blue under visible light illumination than pristine TiO_2_ [3]. Similarly, Wang et al. developed B-doped g-C_3_N_4_/black TiO_2_ Z-Scheme heterojunctions with enhanced charge separation efficiency and increased tetracycline sorption ability [4]. Heterojunctions with abundant defects and a bandgap of 2.13 eV could degrade 65% tetracycline hydrochloride in 30 min in the presence of visible light [4].

Some applications in bioactive coatings have also been highlighted. Chifor et al. deposited TiO_2_ nanoparticles on titanium foil together with Au/Ag nanoparticles and lysozyme for dental implants [5]. The metal-modified TiO_2_ coatings demonstrated high antimicrobial activity against M. lysodeicticus, while the bare TiO_2_ could maintain enzymatic activity [5]. Using a simple molten-salt method, Ngo et al. synthesized rutile TiO_2_ microrods with uniform sizes [6]. Oxygen vacancies were generated in rutile TiO_2_, leading to varied photoluminescence emission properties from the wavelength of 400 to 900 nm [6]. Hu et al. prepared Ti^3+^-doped TiO_2_ nanospindles with an enhanced visible light photocatalytic activity using a facile solvothermal process [7]. The crystallinity, morphologies, and photodegradation efficiency of rhodamine B of Ti^3+^/TiO_2_ were severely influenced by the amount of triethanolamine in the synthetic process [7]. Based on the reduced graphene oxide/mesoporous TiO_2_ nanotube heterostructures (rGO/TiO_2_), Li et al. constructed efficient charge migration paths to significantly improve photocatalytic activity [8]. With much promoted charge transfer efficacy, the rGO/TiO_2_ heterojunction demonstrated decent photocatalytic H_2_ evolution efficiency of ca. 933 μmol/g/h in the presence of sunlight [8].

In addition, two review articles highlighting various aspects of TiO_2_-based materials in the research area of photocatalysis have also been included in this Special Issue, providing an overview of the current developments of TiO_2_ nanoparticles for varied applications. Liao et al. summarized the recent progress in black TiO_2_ nanomaterials, including the recently developed techniques for the preparation of black TiO_2_, element doping, metal modification, heterojunction formation, and the corresponding photocatalytic applications [9]. They also outlined different techniques regarding photocatalytic water splitting and pollutant removal using black TiO_2_-based nanomaterials, as well as challenges and opportunities for energy conversion [9]. To analyze the safety and complexity of Ti oxides in disinfection and bioimplants and provide useful information about residual and dark catalysis, Querebillo reviewed the advances in TiO_2_ catalysis and the formation mechanism of reactive oxygen species w/o light irradiation and discussed the microbicidal effects of Ti oxides and prospects for bioimplant utilization [10].

We would like to thank all the authors and reviewers for their contributions to this Special Issue. We also hope that the publications presented here generate interest and inspiration for their designs, concepts, and conclusions and can unveil new perspectives in the field of nanomaterials and catalysis.
